# FUS-ALS mutants alter FMRP phase separation equilibrium and impair protein translation

**DOI:** 10.1126/sciadv.abf8660

**Published:** 2021-07-21

**Authors:** Nicol Birsa, Agnieszka M. Ule, Maria Giovanna Garone, Brian Tsang, Francesca Mattedi, P. Andrew Chong, Jack Humphrey, Seth Jarvis, Melis Pisiren, Oscar G. Wilkins, Micheal L. Nosella, Anny Devoy, Cristian Bodo, Rafaela Fernandez de la Fuente, Elizabeth M. C. Fisher, Alessandro Rosa, Gabriella Viero, Julie D. Forman-Kay, Giampietro Schiavo, Pietro Fratta

**Affiliations:** 1 Department of Neuromuscular Diseases, UCL Queen Square Institute of Neurology, London WC1N 3BG, UK.; 2 UK Dementia Research Institute, University College London, London WC1E 6BT, UK.; 3 Department of Biology and Biotechnology Charles Darwin, Sapienza University of Rome, P.le A. Moro 5, 00185 Rome, Italy.; 4 Center for Life Nano Science, Istituto Italiano di Tecnologia, Viale Regina Elena 291, 00161 Rome, Italy.; 5 Program in Molecular Medicine, The Hospital for Sick Children, Toronto, ON M5G 0A4, Canada.; 6 Department of Biochemistry, University of Toronto, Toronto, ON M5S 1A8, Canada.; 7 Institute of Biophysics, CNR, Trento, Italy.; 8 The Francis Crick Institute, London NW1 1AT, UK.; 9 Maurice Wohl Clinical Neuroscience Institute, King’s College London, London SE5 9RT, UK.; 10 MRC Centre for Neuromuscular Disease, Queen Square, London WC1N 3BG, UK.

## Abstract

FUsed in Sarcoma (FUS) is a multifunctional RNA binding protein (RBP). FUS mutations lead to its cytoplasmic mislocalization and cause the neurodegenerative disease amyotrophic lateral sclerosis (ALS). Here, we use mouse and human models with endogenous ALS-associated mutations to study the early consequences of increased cytoplasmic FUS. We show that in axons, mutant FUS condensates sequester and promote the phase separation of fragile X mental retardation protein (FMRP), another RBP associated with neurodegeneration. This leads to repression of translation in mouse and human FUS-ALS motor neurons and is corroborated in vitro, where FUS and FMRP copartition and repress translation. Last, we show that translation of FMRP-bound RNAs is reduced in vivo in FUS-ALS motor neurons. Our results unravel new pathomechanisms of FUS-ALS and identify a novel paradigm by which mutations in one RBP favor the formation of condensates sequestering other RBPs, affecting crucial biological functions, such as protein translation.

## Teaser

Cytoplasmic mislocalization of FUS-ALS mutants determines aberrant FMRP condensates and protein synthesis repression.

## INTRODUCTION

The fate of mRNAs in the cytoplasm, including their localization, stability and translation, is regulated by RNA binding proteins (RBPs). These RBPs often contain low-complexity regions (LCRs) that promote their phase separation into biomolecular condensates. RNA transport granules and stress granules are two examples of biomolecular condensates and their dynamics, such as trafficking, assembly and disassembly, impact on the availability, localization, and translation of bound RNAs.

FUsed in Sarcoma (FUS) is an LCR-containing RBP involved in various aspects of RNA metabolism. Under physiological conditions, FUS is predominantly localized in the nucleus where it is involved in transcription, mRNA processing, and miRNA biogenesis (*1*); however, low levels of the protein are also present in the cytoplasm (*2*). FUS mutations are causative of amyotrophic lateral sclerosis (ALS), a relentless neurodegenerative disorder in which the loss of motor neurons (MNs) leads to a progressive impairment of the neuromuscular system. Most FUS mutations disrupt the C-terminal nuclear localization signal (NLS), leading to a nuclear depletion and a cytoplasmic mislocalization of FUS (*3*). The loss of FUS nuclear RNA processing functions, however, is not sufficient to induce neurodegeneration on its own, drawing attention to the cytoplasmic roles of FUS and how these are affected by its increased cytoplasmic levels, which ultimately result in cytoplasmic neuronal aggregates in patient post mortem tissue (*4*, *5*).

The role of FUS in the cytoplasm and the functional consequences of ALS-linked mutations are poorly understood; however, an increasing number of studies suggest that FUS liquid-liquid phase separation (LLPS) properties play a crucial role in its cytoplasmic gain of function (*6*–*8*). Under physiological conditions, the propensity of FUS to undergo LLPS in the cytoplasm is limited by its low concentration; in contrast, the increased cytoplasmic localization of FUS mutants favors the transition to a phase-separated state. Moreover, decreased interaction with the nuclear import factor and chaperone TNPO1, posttranslational modifications and intrinsic properties given by ALS mutations all contribute to the formation of cytoplasmic FUS condensates (*6*, *7*, *9*, *10*), the biology and composition of which are, to date, poorly understood. Potential disease mechanisms have been identified in various overexpression systems (*7*, *11*), although it remains unclear how well these model the physiological setting, where the expression of FUS, as well as most RBPs, is finely tuned both by auto- and cross-regulatory mechanisms (*12*).

Here, we use mouse and human models with endogenous expression of FUS-ALS mutations and show that mutant FUS forms cytoplasmic condensates containing the RBP fragile X mental retardation protein (FMRP). FUS and FMRP repress de novo protein synthesis in vitro and in MNs. We find that this is not caused by direct interaction with the translational machinery; instead, our data support a model whereby FUS and FMRP condensates have an antagonistic role to translation, resulting in decreased ribosome occupancy of FMRP mRNA targets. Our findings highlight how mutant FUS can posttranscriptionally alter RBP dynamics and function.

## RESULTS

### FUS-ALS mutants increase axonal FMRP condensates

**Fig. 1:**
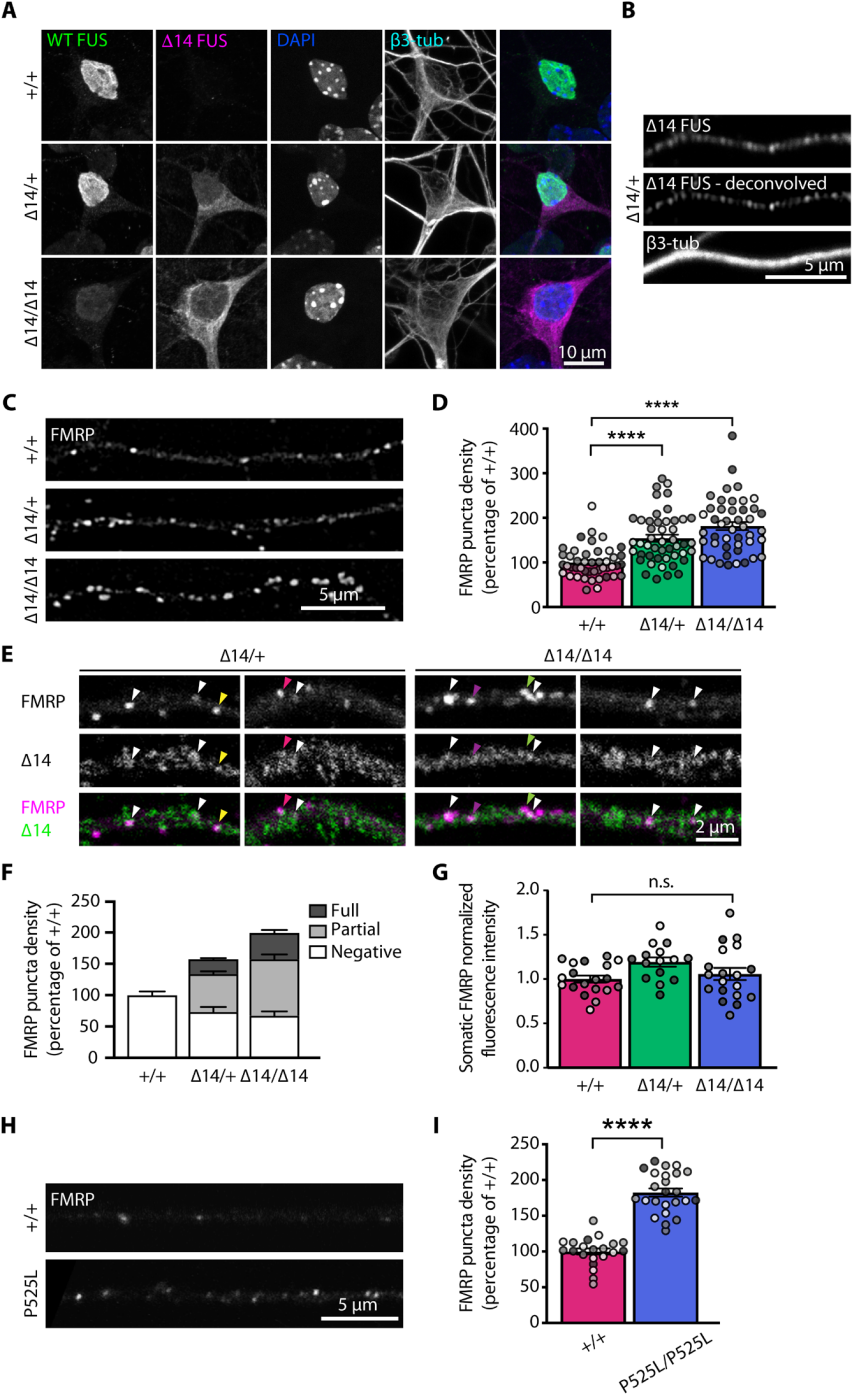
FUS mutants induce increased FMRP puncta density in MN axons. (**A**) Representative images of primary MNs at 5 days in vitro (DIV 5). Wild-type (WT) FUS (green), detected with a C-terminal antibody, is primarily localized in the nucleus in *Fus^+/+^* and *Fus*^∆*14/+*^ neurons. ∆14 FUS (magenta) is enriched in the cytoplasm of *Fus*^∆*14/+*^ and *Fus*^∆*14/*∆*14*^ neurons. Nuclei are labeled with 4′,6-diamidino-2-phenylindole (DAPI) (blue), and β3-tubulin (cyan) is used as a neuronal marker. Low-intensity wild-type FUS-positive nuclear staining in *Fus*^∆*14/*∆*14*^ neurons is due to antibody cross-reactivity with another FET protein, likely EWSR1. (**B**) ∆14 FUS distribution in a *Fus*^∆*14/+*^ MN axon. ∆14 FUS signal detected by confocal microscopy (top) and the deconvoluted signal (middle). Neurons were grown in microfluidic devices, and β3-tubulin is used to identify axons. (**C**) Representative deconvolved images of FMRP axonal puncta in *Fus^+/+^*, *Fus*^∆*14/+*^, and *Fus*^∆*14/*∆*14*^ MNs grown in microfluidic chambers (MFCs) (DIV 8). (**D**) Quantification of axonal FMRP puncta density in *Fus^+/+^*, *Fus*^∆*14/+*^, and *Fus*^∆*14/*∆*14*^ MNs (*n* = 4, axons = 45 to 47, ****P < 0.0001; Kruskal-Wallis, followed by Dunn’s post hoc test). (**E**) Representative images showing axonal FMRP puncta either fully (white arrowheads) or partially (colored arrowheads) positive for ∆14 FUS in *Fus*^∆*14/+*^ and *Fus*^∆*14/*∆*14*^ MNs. (**F**) Segmentation of FMRP puncta density into fully ∆14 positive and partially ∆14 positive and negative. (**G**) Quantification of somatic FMRP fluorescence in *Fus^+/+^*, *Fus*^∆*14/+*^, and *Fus*^∆*14/*∆*14*^ HB9::GFP (green fluorescent protein)–positive MNs (*n* = 4, MNs = 15 to 19). (**H**) Representative images of FMRP axonal puncta in *FUS^+/+^* and *FUS^P525L/P525L^* iPSC-derived MNs grown in MFCs. (**I**) Quantification of axonal FMRP puncta density in *FUS^+/+^* and *FUS^P525L/P525L^* iPSC-derived MNs as shown in (F) (*n* = 4, axons = 21 to 24; ****P < 0.0001, Student’s *t* test). Independent experiments are visualized in different shades of gray in the graphs. n.s., not significant.

To investigate the consequences of ALS-causative mutations on FUS cytoplasmic function, we used the ∆14 FUS knockin mouse model (*13*), in which a mutation causing aggressive and early-onset ALS (*14*) leads to skipping of exon 14 and a frameshift in exon 15. This frameshift leads to the loss of most of the RGG3 domain, the complete deletion of the NLS, and the formation of a unique C-terminal peptide sequence (fig. S1A), allowing the generation of mutant-specific antibodies (*13*). Similarly to *Fus^−/−^* mice (*4*), *Fus*^∆*14/*∆*14*^ mice die perinatally on a congenic background, likely because of respiratory failure. To investigate the function of ∆14 FUS in MNs, as well as its dosage-dependent effects, we used both heterozygous and homozygous primary embryonic MNs. While wild-type FUS has a predominantly nuclear localization in both *Fus^+/+^* and *Fus*^∆*14/+*^ MNs, ∆14 FUS is enriched in the cytoplasm of *Fus*^∆*14/+*^ and *Fus*^∆*14/*∆*14*^ neurons, is distributed across cell body and neurites (Fig. 1, A and B, and fig. S1, B to D), and can be detected in a punctate, condensate-like form (Fig. 1, A and B, and fig. S1B). Notably, ∆14 FUS expression in *Fus*^∆*14/+*^ MNs does not induce the mislocalization of wild-type FUS (Fig. 1A and fig. S1, B and C).

FUS interacts with several other RBPs, including FMRP and Survival Motor Neuron (SMN), two well-characterized RBPs that, similarly to FUS, are present in cytoplasmic RNA granules and are strongly linked to neurological diseases (*15*–*20*). In addition, FMRP has been detected in FUS-positive inclusions in post mortem tissue (*15*). This prompted us to test whether physiological expression of mutant FUS could affect these RBPs. FMRP and SMN interact with both wild-type and ∆14 FUS (fig. S2A). To investigate their localization, we focused on MN axons where both RBPs are incorporated into RNA transport granules. The density of axonal FMRP puncta was increased in primary *Fus*^∆*14/+*^ and *Fus*^∆*14/*∆*14*^ MNs compared to controls in a mutant FUS dosage-dependent manner (Fig. 1, C and D, and fig. S2B; normalized axonal FMRP puncta density in *Fus^+/+^* = 100 ± 5.2, *Fus*^∆*14/+*^ = 154.2 ± 8.2, and *Fus*^∆*14/*∆*14*^ = 181.5 ± 9.2, ****P < 0.0001). In contrast, no significant changes in the somatic intensity of FMRP staining or in the total FMRP expression in MN cultures were detected (Fig. 1G and fig. S2, D to F; somatic FMRP fluorescence intensity in *Fus^+/+^* = 1 ± 0.04, *Fus*^∆*14/+*^ = 1.2 ± 0.05, and *Fus*^∆*14/*∆*14*^ = 1.0 ± 0.07; FMRP levels in cultured MN lysates *Fus^+/+^* = 1.9 ± 0.45, *Fus*^∆*14/+*^ = 1.9 ± 0.4, and *Fus*^∆*14/*∆*14*^ = 1.7 ± 0.4). Puncta size was also unaffected (fig. S2C; average FMRP axonal puncta size in *Fus^+/+^* = 0.26 ± 0.01 μm^2^, *Fus*^∆*14/+*^ = 0.28 ± 0.01 μm^2^, and *Fus*^∆*14/*∆*14*^ = 0.29 ± 0.01 μm^2^).

*FUS* P525L is a well-described mutation that impairs the PY-NLS, leading to the cytoplasmic mislocalization of FUS (fig. S2G). Similar to the mouse primary MNs, we also observed an 80% increase in FMRP axonal puncta in human induced pluripotent stem cell (iPSC)–derived *FUS^P525L/P525L^* MNs (normalized axonal FMRP puncta density in isogenic control = 100 ± 4.4, *FUS^P525L/P525L^* = 182.5 ± 5.8, ****P < 0.0001; Fig. 1, H and I). FMRP puncta colocalized with FUS in both primary and iPSC-derived MNs (Fig. 1, E and F, and fig. S2H), with ~60% of FMRP puncta either fully or partially positive for mutant FUS (percentage of ∆14 FUS-positive FMRP puncta: *Fus*^∆*14/+*^ axons, 16.3% full overlap and 39.1% partial overlap; *Fus*^∆*14/*∆*14*^ axons, 20.6% full overlap and 45.4% partial overlap; Fig. 1, E and F), suggesting a primary role for FUS in altering FMRP dynamics. We found minimal alterations in FMRP puncta density in FUS knockout axons (fig. S2, I and J; normalized axonal FMRP puncta density in *Fus^+/+^* = 100 ± 4.1, *Fus^+/−^* = 77.26 ± 5.5, and *Fus^−/−^* axons = 85.9 ± 5.7), supporting that the increase in axonal FMRP puncta is caused by a gain of function of mutant FUS.

ALS-associated FUS mutants alter SMN dynamics, particularly its localization to nuclear gems (*4*, *16*–*18*). We therefore investigated whether mutant FUS expression also affects SMN distribution along axons. We found that SMN puncta density was unaltered in *Fus*^∆*14/+*^ and *Fus*^∆*14/*∆*14*^ MN axons compared to *Fus^+/+^* axons (fig. S2, K and L; normalized axonal SMN puncta density in *Fus^+/+^* = 100 ± 5.2, *Fus*^∆*14/+*^ = 101.1 ± 5.9, and *Fus*^∆*14/*∆*14*^ = 122.0 ± 7.9), suggesting that only a subset of FUS-binding RBPs, such as FMRP, are affected by mutant FUS.

To assess whether a general impairment in axonal transport could account for the altered FMRP distribution, we analyzed the density of lysosome-associated membrane protein 1 (LAMP1)–positive organelles in MN axons. Mutant FUS expression did not significantly affect the density of these structures within this compartment (fig. S3, A and B; normalized axonal LAMP1 puncta density in *Fus^+/+^* = 100 ± 6.5, *Fus*^∆*14/+*^ = 112.6 ± 6.22, and *Fus*^∆*14/*∆*14*^ = 123.2 ± 8.1). Moreover, we analyzed the axonal transport of both signaling endosomes and mitochondria, and we found it to be unaffected in ∆14 FUS MNs at 7 days in vitro (fig. S3, C to E; signaling endosome average track velocity in *Fus^+/+^* = 1.4 ± 0.08 μm/s, *Fus*^∆*14/+*^ = 1.5 ± 0.1 μm/s, and *Fus*^∆*14/*∆*14*^ = 1.4 ± 0.09 μm/s; time spent pausing in *Fus^+/+^* = 17.1 ± 1.9%, *Fus*^∆*14/+*^ = 11.9 ± 0.9%, and *Fus*^∆*14/*∆*14*^ = 13.61 ± 1.4%; mitochondria average velocity in *Fus^+/+^* = 0.7 ± 0.06 μm/s, *Fus*^∆*14/+*^ = 0.6 ± 0.05 μm/s, and *Fus*^∆*14/*∆*14*^ = 0.6 ± 0.06 μm/s), in agreement with recently published data (*21*).

### FMRP partitions into FUS condensates

**Fig. 2:**
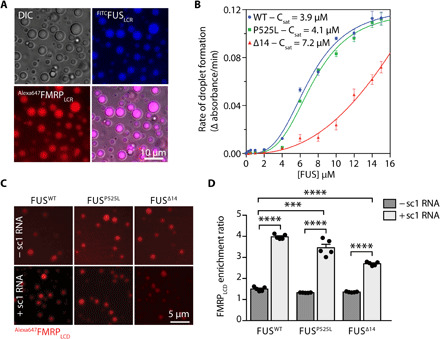
Recombinant FUS promotes FMRP LLPS. (**A**) In vitro co-LLPS assay of ^FITC^FUS_LCR_ with ^Alexa647^FMRP_LCR_ in the presence of sc1 RNA. A total of 50 μM of each protein and 1 μM of sc1 RNA were used (*n* = 3). DIC, differential interference contrast. (**B**) Phase separation propensities of different FUS constructs are determined by the change in turbidity as a function of time. Each point represents the mean rate of turbidity change, and error bars represent the SEM (*n* = 3). (**C**) Representative images of an in vitro co-LLPS assay showing the partitioning of ^Alexa647^FMRP_LCR_ into wild-type, P525L, or ∆14FUS mutants in the presence or absence of sc1 RNA. FUS (10 μM) phase separation was induced by TEV protease (0.5 μM) cleavage before the addition of ^Alexa647^FMRP_LCR_ (1 μM) and sc1 RNA (0.5 μM). Scale bar, 5 μm. (**D**) Quantification of ^Alexa647^FMRP_LCR_ enrichment into FUS^WT^, FUS^P525L^, and FUS^∆14^ droplets as shown in (C) (*n* = 5; ***P < 0.001 and ****P < 0.0001. One-way analysis of variance (ANOVA), followed by Tukey’s multiple comparisons test).

To further explore how mutant FUS induces FMRP granules, we asked whether the two proteins could cophase separate in vitro. We performed an in vitro LLPS assay in which the disordered N-terminal region of FUS conjugated with fluorescein isothiocyanate (FITC) (^FITC^FUS_LCR_) was coincubated with the disordered C-terminal region of FMRP conjugated with Alexa Fluor 647 (^Alexa647^FMRP_LCR_), in the presence of sc1, a G-quadruplex–forming RNA known to bind FMRP (*22*, *23*). The observed droplets were positive for both ^FITC^FUS_LCR_ and ^Alexa647^FMRP_LCR_ (Fig. 2A), evidence that the FUS disordered N-terminal region phase separates with sc1 RNA and FMRP.

To analyze the partitioning of FMRP into droplets of full-length FUS, we generated wild-type, P525L, and ∆14 recombinant FUS, fused to maltose binding protein (MBP) to enhance solubility and circumvent the strong propensity of recombinant FUS to aggregate (fig. S4A). Upon cleavage of the MBP tag by Tobacco Etch Virus (TEV) protease, all FUS proteins phase-separated in vitro as detected by turbidity measurements (Fig. 2B). FUS^Δ14^ displayed a lower LLPS propensity compared to FUS^WT^ and FUS^P525L^, likely due to the loss of the C-terminal RG/RGG region (fig. S4C). To investigate FMRP incorporation into FUS condensates, we added ^Alexa647^FMRP_LCR_ to preformed FUS droplets in the presence or absence of sc1 RNA. FMRP was equally enriched in FUS^WT^, FUS^P525L^, and FUS^Δ14^ condensates in the absence of sc1 RNA, and sc1 RNA increased the enrichment of ^Alexa647^FMRP_LCR_ in these condensates for all FUS proteins (^Alexa647^FMRP_LCR_ enrichment ratio in FUS^WT^ = 1.5 ± 0.04, FUS^WT^ + sc1 = 4.0 ± 0.05, FUS^P525L^ = 1.3 ± 0.004, FUS^P525L^ + sc1 = 3.5 ± 0.17, FUS^Δ14^ = 1.4 ± 0.008, and FUS^Δ14^ + sc1 = 2.7 ± 0.04; ***P < 0.001 and ****P < 0.0001; Fig. 2, C and D). These results show that FMRP partitions into FUS droplets via protein-protein and protein-RNA interactions. The finding that wild-type and mutant FUS do not significantly differ is not unexpected as wild-type FUS is physiologically prevalently nuclear, while these assays recapitulate the behavior of the proteins when present in the same compartment.

To further analyze FUS-induced FMRP sequestration in a cellular environment, we took advantage of the ability of mutant FUS to generate intracellular condensates upon overexpression (*7*). Overexpression of either NLS-lacking FUS (^mCherry^FUS^513x^) or mutant FUS (^Flag^FUS^P525L^) in HeLa cells induced the formation of large, cytoplasmic FUS-positive condensates (fig. S5A, left), which led to the sequestration of endogenous FMRP (fig. S5A). We found that brief (~18-hour) overexpression of ^mCherry^FUS^513x^ led to the presence of small FMRP puncta decorating larger FUS condensates (fig. S5A, top), as well as condensates with a more homogeneous distribution of FMRP and FUS (fig. S5A, middle), possibly reflecting different phases of incorporation or a multiphasic behavior reminiscent of FMRP-Caprin1 condensates (*24*). Overexpression of ^Flag^FUS^P525L^ in primary MNs also led to the formation of distinct FUS condensates that were positive for endogenous FMRP (fig. S5; arrowheads indicate FMRP-positive FUS puncta).

Because FUS overexpression may induce the formation of stress granules, the increase in FMRP condensates could be due to cellular stress rather than a direct consequence of cytoplasmic mutant FUS. To address this, we generated mouse embryonic fibroblasts (MEFs) from our ∆14 FUS mouse model, which endogenously expresses mutant FUS. When we looked at endogenous FMRP, we found that the presence of mutant FUS led to a dose-dependent increase in the number of cells with spontaneous FMRP condensates (>0.5 μm^2^) compared to controls (fig. S5, C to F; percentage of MEFs with FMRP puncta: *Fus^+/+^* = 46.2 ± 2.1%, *Fus*^∆*14/+*^ = 55.8 ± 3.6%, and *Fus*^∆*14/*∆*14*^ = 63.8 ± 5.4%; **P < 0.05). The large majority of these puncta were negative for stress granule markers, such as Ras-GAP SH3 domain binding protein 1 (G3BP1) (fig. S5, C and D; percentage of MEFs with G3BP1 puncta *Fus^+/+^* = 1.5 ± 0.6%, *Fus*^∆*14/+*^ = 0.8 ± 0.4%, *Fus*^∆*14/*∆*14*^ = 1.7 ± 0.7%). Together, these findings further indicate that cytoplasmic mutant FUS increases localization of FMRP in cytoplasmic FUS condensates in neuronal and non-neuronal cells and provides evidence that the presence of FMRP in these structures is not a secondary effect triggered by cellular stress.

### FUS and FMRP repress translation in vitro

**Fig. 3:**
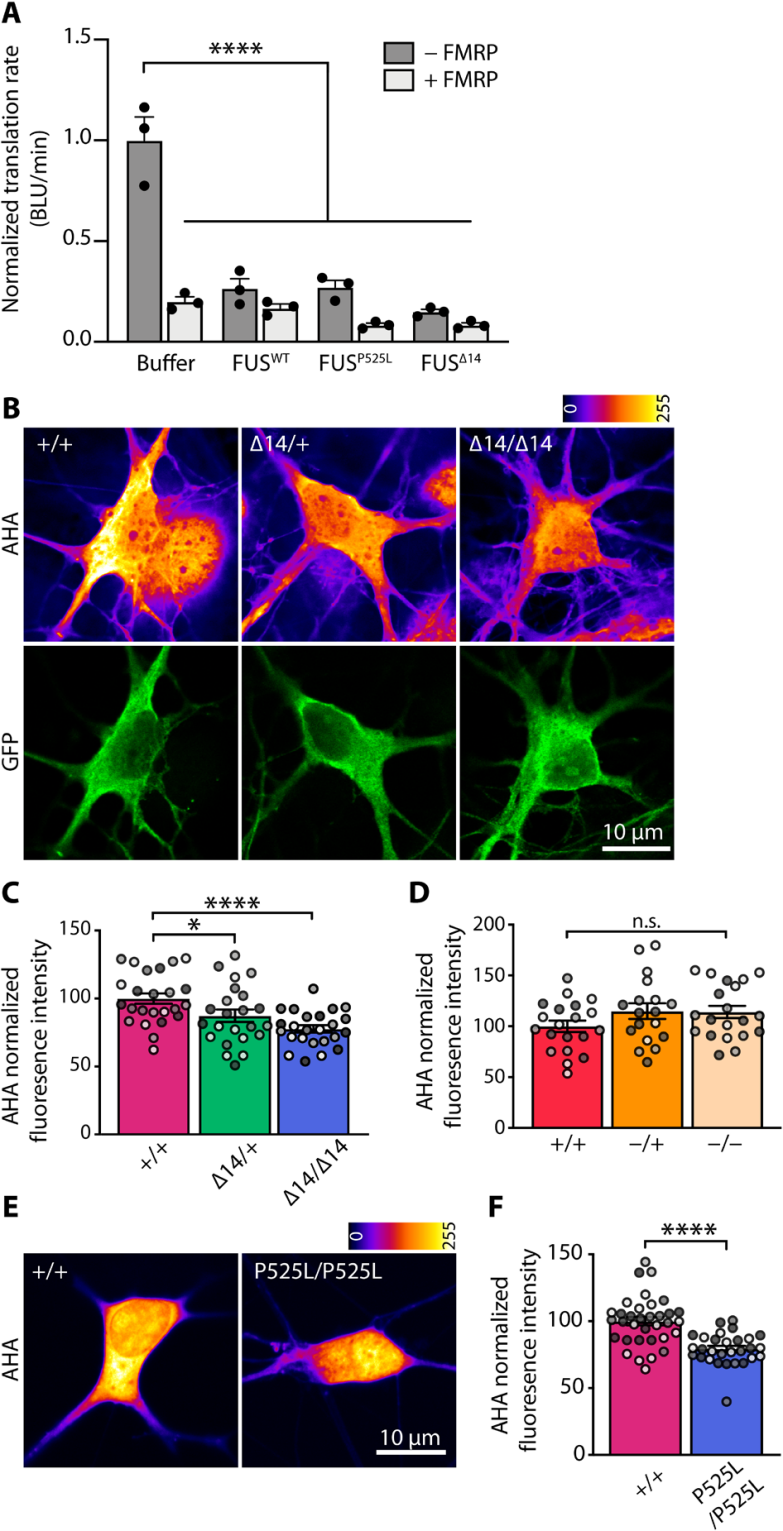
Mutant FUS represses translation in vitro and in cultured MNs. (**A**) Recombinant FUS^WT^, FUS^P525L^, and FUS^Δ14^ (10 μM) phase separation was induced by TEV protease (0.5 μM) cleavage. Proteins were added to an in vitro rabbit reticulocyte translation system with luciferase mRNA in the absence or presence of FMRP (10 μM). Change in bioluminescence (BLU) rate is used as a reporter for translational activity. All results were normalized to buffer control (+TEV) (*n* = 3, ****P < 0.0001; one-way ANOVA, followed by Sidak’s multiple comparisons test). (**B**) Representative images of primary *Fus^+/+^*, *Fus*^∆*14/+*^, and *Fus*^∆*14/*∆*14*^ MNs metabolically labeled using the methionine analog AHA (2 mM, 30 min) and click chemistry. AHA labeling is visualized using the LUT fire (top), and MNs are identified by GFP expression under the HB9 promoter (bottom). (**C**) Quantification of the AHA labeling as shown in (C). Mean fluorescence intensity values are normalized to *Fus^+/+^* (*n* = 4, MNs = 27 to 28; one-way ANOVA, followed by Dunnett’s multiple comparisons test, *P < 0.05 and ****P < 0.0001). (**D**) Quantification of translation assays carried out in *Fus^+/+^*, *Fus^+/−^*, and *Fus^−/−^* MNs. Mean fluorescence intensity values are normalized to *Fus^+/+^* (*n* = 3, MNs = 18 to 20). (**E**) Representative images of isogenic control (*FUS^+/+^*) and *FUS^P525L/P525L^* iPSC-derived MNs metabolically labeled with AHA (2 mM, 30 min). (**F**) Quantification of the effect in (E). Fluorescence values are normalized to *FUS^+/+^* MNs (*n* = 3, MNs = 29 to 34; Student’s *t* test, ****P < 0.0001). Independent experiments are visualized in different shades of gray in the graphs.

Biomolecular condensates are thought to antagonize translation (*25*, *26*), and FMRP phase separation correlates with translation inhibition (*23*). To test whether FUS condensates can directly affect protein synthesis, we took advantage of an in vitro translation assay in which recombinant MBP-FUS fusion proteins were directly added to a commercial rabbit reticulocyte cytoplasmic extract and its phase separation was induced by TEV protease cleavage. Quantification of the bioluminescence of the firefly luciferase, as a reporter for the translation of its mRNA, showed that all FUS proteins significantly suppressed translation, and addition of FMRP_LCR_ induced a further decrease in luciferase translation (normalized bioluminescence per minute in buffer control = 1 ± 0.12, buffer + FMRP = 0.2 ± 0.02, FUS^WT^ = 0.27 ± 0.05, FUS^WT^ + FMRP = 0.17 ± 0.02, FUS^P525L^ = 0.27 ± 0.03, FUS^P525L^ + FMRP = 0.08 ± 0.01, FUS^Δ14^ = 0.15 ± 0.01, FUS^Δ14^ + FMRP = 0.08 ± 0.01; ****P < 0.0001; Fig. 3A). These results support FUS having a repressive role on protein synthesis and that its mislocalization to the cytoplasm and not the mutations per se is key to driving this gain-of-function mechanism. FMRP has an additive, although not significant, effect on translation inhibition.

### Cytoplasmic FUS represses translation in MNs

Two reports have recently shown that mutant FUS overexpression can impair protein synthesis in neurons (*7*, *27*), but whether this also occurs at physiological FUS expression is not currently known. We analyzed de novo protein synthesis in primary motoneuronal cultures, using the methionine analog l-azidohomoalanine [(AHA) 2 mM, 30-min incubation] in combination with click chemistry. We found that, compared to wild-type controls, AHA labeling was reduced by 13% in *Fus*^∆*14/+*^ and by 20% in *Fus*^∆*14/*∆*14*^ primary MNs (average AHA intensity in *Fus^+/+^* MNs 100 ± 3.8, *Fus*^∆*14/+*^ MNs 87.4 ± 4.5, and *Fus*^∆*14/*∆*14*^ MNs 77.7 ± 2.7; *P < 0.05 and ****P < 0.0001; Fig. 3, B and C). As a positive control, we showed that pretreatment with the translation inhibitor anisomycin (40 μM, 20 min) reduced the AHA signal by 80%, indicating the specificity of the labeling (fig. S6, B and C). In contrast, in FUS knockout MNs, no significant effect on de novo protein synthesis was detected (average AHA intensity in *Fus^+/+^* MNs 100 ± 5.5, *Fus^+/−^* MNs 115 ± 7.7, and *Fus^−/−^* MNs 114 ± 6.0; Fig. 3D and fig. S6A), indicating that inhibition of translation is due to a gain of function of mutant FUS.

To test whether this deficit was conserved in human models of FUS-ALS, we performed the same assay in iPSC-derived MNs carrying the common ALS-associated NLS mutation P525L and compared them to isogenic controls. Similarly to mouse MNs, AHA labeling was decreased by 20% in mutant FUS human MNs (isogenic control 100 ± 3.1 and *FUS^P525L/P525L^* 79.4 ± 2.2; ****P < 0.0001; Fig. 3, E and F), underlining that translation inhibition stems from FUS cytoplasmic mislocalization and is not mutation specific.

### Mutant FUS does not alter translation by association with polysomes

Despite its low cytoplasmic levels, wild-type FUS binds proteins of both the small and large ribosomal subunits (*28*), suggesting that it may interact with assembled ribosomes. Given the increase in cytoplasmic levels of mutant FUS, we asked whether association with ribosomes could account for the observed changes in translation. To investigate this, we performed polysome cosedimentation assays, where separation of the heavier polysomal fractions from monosomes (80*S*), the individual ribosomal subunits (60*S* and 40*S*), and the lighter free cytosolic complexes (fig. S7Ai) allows the analysis of the association of specific proteins with the translation components. As expected, most of wild-type FUS cosedimented with free cytosolic complexes, but significant levels also cosedimented with polysomes in both *Fus^+/+^* and *Fus*^∆*14/+*^ samples (fig. S7Aii). However, ∆14 FUS did not cosediment with polysomal fractions, despite its large cytoplasmic localization (fractions 8 to 11; fig. S7Aii), and was only present in the lighter part of the gradient, which contains subpolysomal components, in *Fus*^∆*14/+*^ and *Fus*^∆*14/*∆*14*^ samples (fractions 1 to 6; fig. S7Aii).

Because both FMRP and SMN are also known to associate with the translational machinery (*29*, *30*), we examined their cosedimentation profile in the ∆14 FUS model. While both FMRP and SMN interact with ∆14 FUS (fig. S2A), when analyzing their cosedimentation, we found that the distribution of SMN was unaltered by the expression of mutant FUS, whereas FMRP was depleted from the polysomal fractions in both *Fus*^∆*14/+*^ and *Fus*^∆*14/*∆*14*^ samples (fig. S7A). We confirmed the weaker association of FMRP with ribosomes in ∆14 FUS MNs using proximity ligation assays (PLAs) between FMRP and the ribosomal protein RPL26 (normalized FMRP-RPL26 (ribosomal protein L26) PLA puncta in *Fus^+/+^* = 1.0 ± 0.14, *Fus*^∆*14/+*^ = 0.18 ± 0.04, and *Fus*^∆*14/*∆*14*^ = 0.37 ± 0.1; *P < 0.05 and ****P < 0.0001; fig. S7, C and D). We found FUS depletion (*Fus^−/−^*) to have a similar effect on FMRP-ribosome association both in cosedimentation assays (fig. S7B) and in FMRP-RPL26 PLAs (normalized FMRP-RPL26 PLA puncta in *Fus^+/+^* = 1.0 ± 0.12, *Fus^+/−^* = 0.9 ± 0.21, and *Fus^−/−^* = 0.44 ± 0.08; **P < 0.001; fig. S7E).

In summary, these results show that FUS mutations impair its association with polysomes and that wild-type FUS promotes the association of FMRP with the translation machinery. Given that the global level of protein synthesis is not affected in FUS knockout MNs and that polysomal localization of FUS and FMRP is impaired in both knockout and mutant FUS neurons, we conclude that these alterations cannot be the main driver for the translation phenotype observed in Fig. 3, B to F.

### Mutant FUS impairs translation of FMRP target RNAs in vivo

**Fig. 4:**
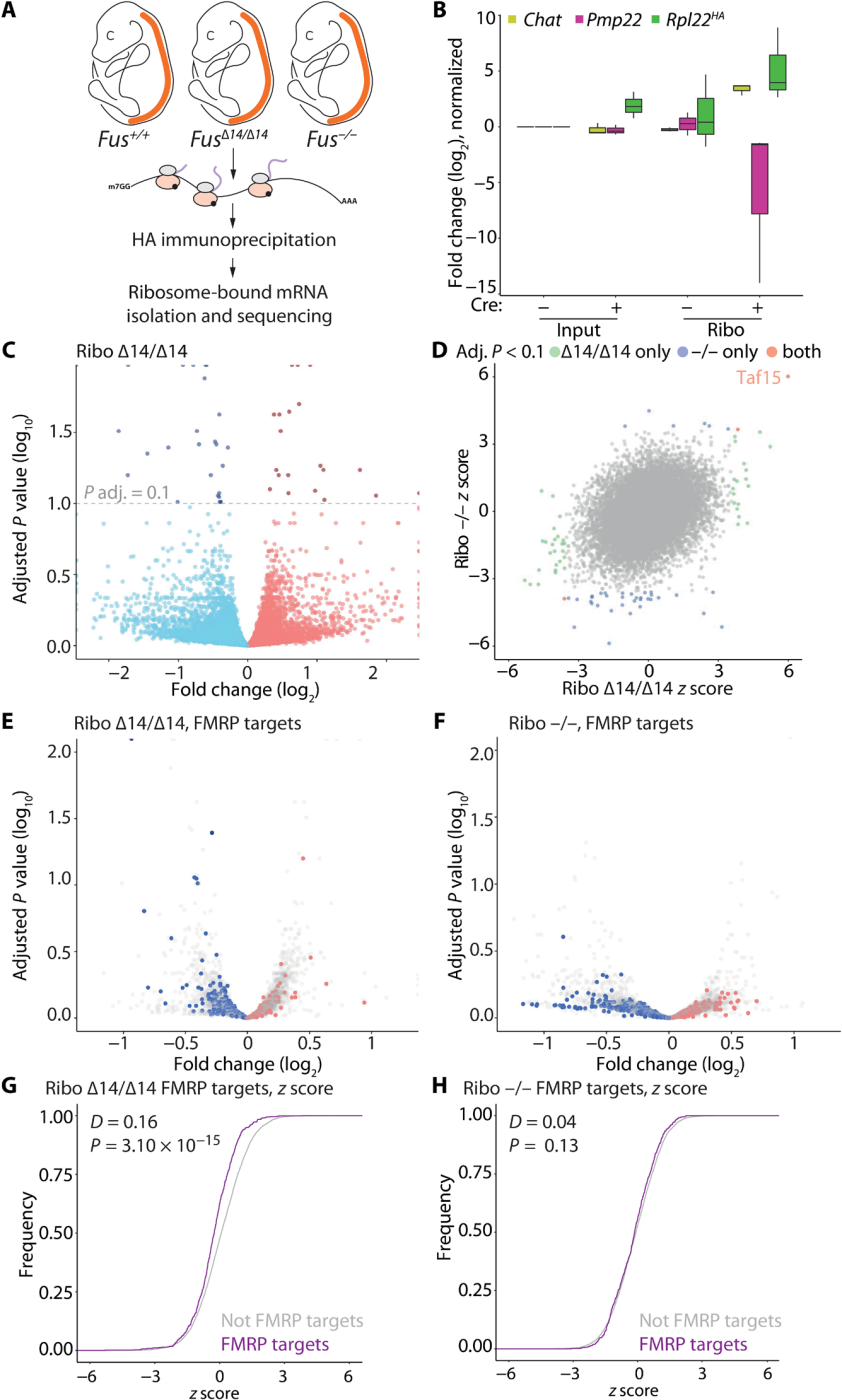
Translation of FMRP-bound genes is decreased in MNs in vivo. (**A**) RiboTag method was used to purify MN-specific, translation-engaged transcripts from embryonic spinal cords (E17.5) of *Fus*^Δ*14/*Δ*14*^ and *Fus^−/−^* and their wild-type littermates. (**B**) qPCR analysis of total spinal cord tissue (input) and HA-tagged, ribosome-associated, MN-specific fraction (Ribo). Expression of MN markers: *Chat* and *Rpl22^HA^* and the glial marker *Pmp22* were measured, *Gapdh* expression was used as housekeeping control (adult spinal cord tissue, 3 months of age, *n* = 3). (**C**) Volcano plot of MN-specific translatome Ribo-*Fus*^Δ*14/*∆*14*^. Blue points, fold change (log_2_) < 0; red points, fold change (log_2_) > 0, genes with fold change (log_2_) > 2.25 or < −2.25 or with adjusted *P* value (−log_10_) < 2 were plotted as infinity (*n* = 5). (**D**) Distribution of *z* scores in the MN-specific translatome Ribo-*Fus*^Δ*14/*Δ*14*^ and Ribo-*Fus^−/−^* shows mutation-specific changes. Green points, adjusted *P* value of <0.1 for *Fus*^Δ*14/*Δ*14*^ only; blue points, adjusted *P* value of <0.1 for *Fus^−/−^* only; and red points, adjusted *P* value of <0.1 for *Fus*^Δ*14/*Δ*14*^ and *Fus^−/−^*. (**E**) Volcano plot of MN-specific translatome Ribo-*Fus*^Δ*14/*Δ*14*^ [filtered by expression (log_10_) base mean < 4.25 and > 2.5, FMRP targets in red and blue, not FMRP targets in gray]. (**F**) Volcano plot of MN-specific translatome Ribo-*Fus^−/−^* filtered by expression (log_10_) base mean < 4.25 and > 2.5, FMRP targets in red and blue, no FMRP targets in gray. (**G**) Cumulative frequency plot of *z* scores of genes in (E) shows a significant decrease of FMRP targets expression in MN-specific, translatome of *Fus*^Δ*14/*Δ*14*^ (Kolmogorov-Smirnov test). (**H**) Cumulative frequency plot of *z* scores of genes shown in (F) shows no change of FMRP targets expression in MN-specific, translatome of *Fus^−/−^* (Kolmogorov-Smirnov test).

We next investigated whether mutant FUS expression could affect the translation of RNAs specifically bound to either FUS or FMRP in vivo. We crossed ∆14 FUS mutant and FUS knockout lines with mice expressing both the Cre-dependent hemagglutinin (HA)–tagged RPL22 ribosomal subunit (*Rpl22^HA^*, RiboTag) and a MN-specific Cre-recombinase (*Chat-Cre*) (*31*). We obtained two triple transgenic lines, *Chat-Cre*/*Fus*^∆*14/*∆*14*^/*Rpl22^HA^* and *Chat-Cre*/*Fus*^−/−^/*Rpl22^HA^*, allowing us to immunopurify ribosome-bound transcripts from MNs within the mouse spinal cord. We refer to ribosome-associated mRNAs (Ribo) as the “translatome” throughout the manuscript (Fig. 4A and fig. S8, A to C). To validate MN specificity of purified RNAs, we performed quantitative polymerase chain reaction (qPCR) experiments that confirmed enrichment for the motor neuronal genes *Chat* and *Rpl22^HA^* and depletion of glial gene *Pmp22* (Fig. 4B).

To identify changes in the translatome caused by the cytoplasmic mislocalization of mutant FUS, we compared changes of Ribo-*Fus*^∆*14/*∆*14*^ versus littermate controls to ones in Ribo*-Fus^−/−^* versus their own littermate controls. We found 21 up-regulated and 26 down-regulated transcripts (adjusted *P* < 0.1) when comparing the Ribo-*Fus*^∆*14/*∆*14*^ translatome to wild-type littermate controls (Fig. 4C and table S1), while the analysis of Ribo-*Fus^−/−^* and wild-type littermate control (fig. S8D and table S1) identified 8 up-regulated and 39 down-regulated transcripts (adjusted *P* < 0.1). The comparison of the *z* score for each gene in the Ribo-*Fus*^∆*14/*∆*14*^ and Ribo-*Fus^−/−^* experiments showed little correlation between the two datasets (Pearson correlation coefficient *r* = 0.28, *P* < 2.2 × 10^−16^). Only 3 transcripts were significantly differentially represented in both Ribo-*Fus*^∆*14/*∆*14*^ and Ribo-*Fus^−/−^*, while others were specific to the translatome of each genotype (3 transcripts with an adjusted *P* < 0.1 in both Ribo*Fus*^∆*14/*∆*14*^ and Ribo*-Fus^−/−^*, 43 transcripts only in Ribo*-Fus*^∆*14/*∆*14*^, and 39 transcripts only in Ribo*-Fus^−/−^*; Fig. 4D and table S2). One of the commonly altered transcripts was *Taf15*, a member of the FET family (along with *Fus* and *Ewsr1*), likely as a compensatory response to FUS nuclear loss. We performed gene ontology (GO) term analysis on Ribo*-Fus*^∆*14/*∆*14*^ and Ribo*-Fus^−/−^* (fig. S8). Mutant FUS caused an up-regulation of genes related to ribosomal biogenesis, which was absent in FUS knockout.

We next asked whether RNAs bound by FUS or by FMRP were affected at the translatome level. We used published and widely used CLIP (individual-nucleotide resolution Cross-Linking and ImmunoPrecipitation) data to select RBP-bound RNAs (*30*, *32*). Because FUS, unlike FMRP, mostly binds pre-mRNA intronic sequences, we selected only transcripts where FUS binds within the mature RNA sequence [5′ untranslated region (5′UTR), CDS (coding sequence), and 3′UTR]. We plotted the distribution of target RNAs (bound by a defined RBP) and non-target RNAs (not bound) (fig. S9B) as a cumulative frequency of their *z* scores and used non-target RNAs with similar expression levels to target RNAs to avoid a bias deriving from gene expression levels (fig. S9 for FUS and fig. S10 for FMRP). In this analysis, a shift of the cumulative frequency curve toward the right indicates an up-regulation of targets in the condition of interest, and a leftward shift indicates a down-regulation (*33*, *34*). The comparison between FUS targets versus non-target controls shows a minor, albeit significant, right shift (up-regulation) of the cumulative distribution in Ribo-*Fus*^∆*14/*∆*14*^ (distance *D* = 0.05; *P* = 1.20 × 10^−5^), while no change was detected in the Ribo-*Fus^−/−^* dataset (*D* = 0.02; *P* = 0.26; fig. S9C). Conversely, when investigating FMRP targets, we found more widespread changes (Fig. 4, E and F, and fig. S10B). We found that FMRP target distribution was down-regulated selectively in Ribo-*Fus*^∆*14/*∆*14*^ (left shift, *D* = 0.16, *P* = 3.10 × 10^−15^; Fig. 4G), while no distribution change was present in Ribo-*Fus^−/−^* (*D* = 0.04, *P* = 0.13; Fig. 4H). This indicates that the presence of mutant FUS in the cytoplasm leads to impaired ribosome association of FMRP targets, supporting the translation inhibition by FUS and FMRP condensates shown in previous assays (Figs. 1 to 3).

GO analysis of down-regulated FMRP targets highlighted terms related to axonal compartments in Ribo-*Fus*^∆*14/*∆*14*^, while these were absent in Ribo-*Fus^−/−^* (fig. S10C; Ribo-*Fus*^∆*14/*∆*14*^: fold change < 0, *P* < 0.05, 33 genes; and fig. S10D; Ribo-*Fus^−/−^*: fold change < 0, *P* < 0.05, 21 genes). We then compared the RiboTag to published mass spectrometry on iPSC-derived FUS P525L MNs used throughout this work (*35*). We found seven human orthologs, with FMRP binding sites at the transcript level, to be down-regulated (*z* score > 1 in both datasets) in both Ribo-*Fus*^∆*14/*∆*14*^ and *FUS^P525L/P525L^* MN mass spectrometry (respective mouse genes: *Dnm1*, *Usp9x*, *Trim2*, *Pld3*, *Foxk2*, *Arfgef1*, and *Tmod2*; table S3). *TRIM2* encodes a ubiquitin ligase that targets the large neurofilament, and *TRIM2* recessive loss-of-function mutations have been found to be causative for hereditary motor axonal neuropathy (*36*).

## DISCUSSION

**Fig. 5:**
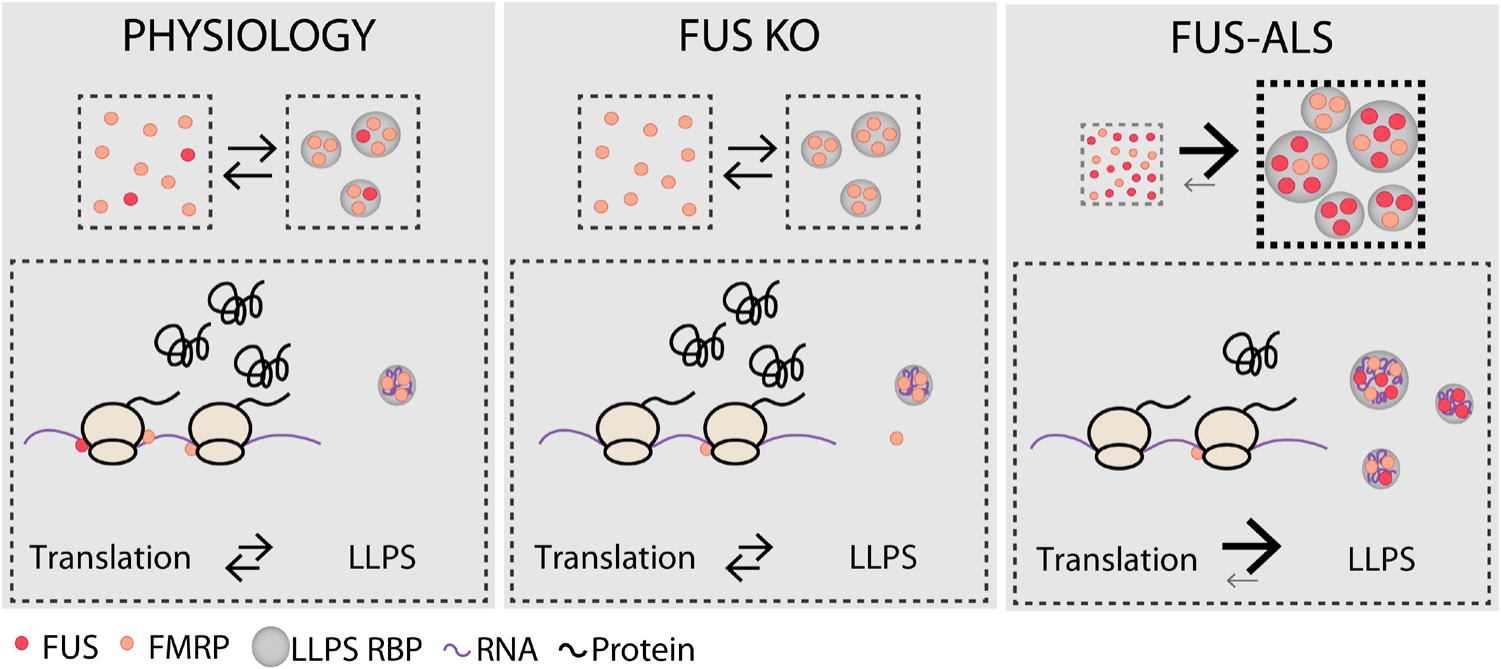
Proposed model of mutant FUS cytoplasmic gain of function. Under control conditions (**left**), low levels of FUS are present in the cytoplasm and the phase separation of FUS and FMRP are at a physiological equilibrium. Loss of FUS (**middle**) results in a reduction of FMRP association with the translational machinery; however, this does not induce significant alterations in FMRP LLPS or global protein translation. In FUS-ALS (**right**), the increased cytoplasmic localization of FUS shifts the LLPS equilibrium of both FUS and FMRP, resulting in an increase in cytoplasmic condensates. This is associated with a depletion of the proteins from the translational machinery and an overall decrease in protein synthesis.

Cytoplasmic mislocalization and nuclear depletion of FUS are hallmarks of FUS-ALS, and the degree of cytoplasmic misplacement induced by different disease-causing mutations correlates with disease severity (*3*). The molecular mechanisms underlying FUS-mediated neuronal toxicity are unclear; however, alterations in phase separation propensities and protein translation have been proposed to have a relevant role (*7*, *27*, *37*, *38*). Moreover, FUS expression levels, similarly to many RBPs, are physiologically highly regulated, and both exogenous overexpression and complete depletion were found to affect numerous cellular processes, causing neurodegeneration and lethality (*4*, *39*–*41*). We investigated the consequences of mutant FUS mislocalization using both mouse and human models, where FUS mutants are expressed at endogenous levels. We analyzed neurons that express mutant FUS both in heterozygosity, as in patients with ALS, and in homozygosity, to better uncover phenotypes. We found that FUS forms condensates where FMRP, another RBP strongly linked to translation regulation and neurodegeneration (*42*–*45*), is sequestered. We show that endogenous expression of FUS mutants is sufficient to impair translation both in mouse and human MNs. Last, we demonstrate that translation of FMRP target RNAs is impaired in vivo in MNs, establishing a pathogenesis paradigm by which mutant FUS impairs translation of specific sets of transcripts by altering the phase behavior of another RBP (Fig. 5).

Recently, numerous studies have demonstrated that FUS can undergo LLPS and that cytoplasmic FUS granules are biomolecular condensates (*7*, *11*). Cytoplasmic condensates have been shown to be involved in various aspects of RNA metabolism. Not only phase separation of specific RBPs is required for the formation of transport granules and stress granules but also the fine tuning of condensate assembly and disassembly is key to controlling RNA availability and, ultimately, translation. In agreement with this, activity-dependent protein translation has been associated with the disassembly of RNA granules and the consequent release of RBP-bound transcripts. Neuronal activity triggers β-actin mRNA granule disassembly (*46*, *47*), associated with activity-dependent protein synthesis; similarly, injury-induced disassembly of G3BP1 granules results in the translation of G3BP1-bound RNAs (*26*).

Mutant ∆14 FUS condensates are present throughout the MN cell body and neurites (Fig. 1, A and B) and are likely to have an impact on RBP dynamics and RNA metabolism before the formation of pathological aggregates seen in post mortem tissue (*5*). The composition of biomolecular condensates is heterogeneous, and the proteins that partition in these structures dictate their biological function. While LCRs of proteins are required for LLPS, RBPs containing these regions have different propensities to phase-separate, and in vitro studies have demonstrated that FUS is particularly prone to undergo this process. Moreover, FUS phase separation can favor the copartitioning of so-called “client proteins” that contain LCRs but would not normally form condensates at physiological concentrations (*48*). FUS condensates could, therefore, act as a scaffolding in which other proteins partition, and the resulting aberrant compartmentalization of these factors could determine alterations in their biological activity. With this in mind, we questioned whether the presence of cytoplasmic FUS condensates could alter the distribution and partitioning of a wider RBP network. Because some FUS-interacting RBPs are also found in FUS inclusions in post mortem tissue or are disrupted in models of FUS-ALS (*4*, *15*, *49*), we decided to analyze the distribution of the two most well-characterized RBPs: FMRP and SMN. When we analyzed the distribution of axonal FMRP, we found a dose-dependent increase in FMRP condensates in mutant FUS primary MNs (Fig. 1, C, D, H, and I) and iPSC-derived MNs, which is not due to alterations in FMRP expression levels but likely reflects a shift in the equilibrium between diffuse FMRP and FMRP granules. We investigated the axonal distribution of these RBPs because they have key roles in RNA transport and axonal function; however, it is likely that the effect is not restricted to this compartment, and we found alterations in the frequency of FMRP condensates also in non-neuronal MEF cells (fig. S5, C and D). Although FMRP granules can be induced by cellular stress, FUS-induced FMRP condensates are negative for stress granule markers (fig. S5, C and D). We therefore propose that the increased FMRP condensate formation is due to a cytoplasmic aberrant function of mutant FUS. Although other cytoplasmic RBPs containing LCRs are likely to copartition within FUS condensates, we did not find any alteration in the distribution of SMN (fig. S2, K and L). Although the expression of mutant FUS may still affect SMN functionality [for example at the nuclear level, as previously reported (*4*)], this indicates that, in addition to protein-protein interaction, other factors are required to promote FUS-induced condensate formation.

We report that expression of FUS mutants at endogenous levels impairs translation, both in physiological models of disease (heterozygous FUS mutant mice) and in MNs carrying homozygous mutations. A recent study, where FUS was overexpressed in human embryonic kidney (HEK) 293 cells, has shown that FUS-mediated translation inhibition was associated with increased cosedimentation of FUS mutants with polysomes (*38*). In our model, however, we find that endogenously expressed mutant FUS does not associate with the translational machinery using biochemical and imaging approaches, a difference that could be due to the overexpression of the protein or to an alternative regulation in HEK293 cells. While FUS-mediated translation regulation has not yet been studied in detail, the role of FMRP as a translation repressor is well established. FMRP inhibits translation through several mechanisms, including polysome binding, miRNA and RNA-induced silencing complex–dependent repression, or binding to translation initiation factors (*43*). More recently, FMRP LLPS has been shown to correlate with translation inhibition in vitro (*23*), and in vivo FMRP granule formation decreases the translation of FMRP-bound synaptic targets (*50*). This adds complexity to the FMRP-dependent regulation of translation and supports a model in which an increase in FMRP condensates could be associated with decreased translation of FMRP targets. To explore how the translation landscape is affected by mutant FUS expression in vivo, we sequenced ribosome-engaged transcripts from MNs. FUS mutations induce transcript expression changes, and we have previously shown that these are comparable to but weaker than FUS knockout (*4*, *51*). However, when we compared the translatome datasets, we found specific changes in the ∆14 FUS compared to the knockout translatome (Fig. 4D), demonstrating that mutant FUS affects the translatome through a gain-of-function mechanism. When we analyzed FMRP target RNAs, we found a selective depletion of these transcripts from the purified ribosomal fractions of our mutant ∆14 FUS model, with no changes in FUS knockout (Fig. 4G). The depletion induced by mutant FUS is notably similar to that described in an FMRP knockout model [fig. S10C; reanalyzed from (*34*); Ribo-*Fmr1^−/y^* *D* = 0.18, *P* = 2.56 × 10^−11^; total RNA *Fmr1^−/y^* D = 0.19, *P* < 2.2 × 10^−16^].

In agreement with FMRP having a concurrent role in FUS-mediated toxicity, FMRP coexpression can rescue FUS-dependent denervation in zebrafish (*15*). However, because FMRP LLPS behavior, rather than expression levels, is affected by mutant FUS expression, and given that overexpression of FMRP itself promotes its LLPS, we believe that it is unlikely that FMRP overexpression could rescue the translation deficit in our model, although it may have some localized effect on specific targets (*52*).

Cytoplasmic mislocalization of FUS also occurs in the absence of disease-associated mutations, both in ALS cases caused by other genetic determinants (*53*) and in cases of frontotemporal lobar degeneration (*54*, *55*). In our in vitro translation assays, we show that wild-type FUS impairs translation in a similar manner to ALS mutants (Fig. 3A). Although FUS-ALS mutations may also affect the biophysical properties of the condensates, such as preventing their dissolution (*7*), and therefore cause a worsening of the phenotype, FUS mislocalization may be sufficient to drive translation repression in these pathologies. Moreover, in our in vitro experiments, FUS^WT^ and FUS^P525L^ have a higher propensity to undergo LLPS compared to FUS^∆14^ (Fig. 2B), which is likely due to the lack of the C-terminal RGG/RG region (fig. S4C). Because both ALS-mutants cause a very aggressive disease in patients and NLS-lacking FUS mutants, such as FUS^∆14^, have a stronger mislocalization compared to NLS missense mutants, such as FUS^P525L^, this highlights how a combination of mislocalization and LLPS behavior can determine FUS cytoplasmic aberrant function and result in comparable cellular toxicity.

In addition to the aforementioned mechanisms, mislocalization of FUS can result in alterations of the protein cellular concentration by affecting its autoregulation and therefore leading to the worsening of its aberrant cytoplasmic functions. Previous studies have shown that FUS-ALS mutations result in increased *Fus* RNA levels (*51*, *56*); we here find *Fus* to be increased also in the mutant translatome dataset. This further supports that the altered transcript expression indeed results in increased FUS protein translation, which, in turn, could be crucial in increasing its cytoplasmic partitioning and create a vicious cycle leading to increased FUS and FMRP condensate formation.

Our results support a model whereby the presence of cytoplasmic mutant FUS-positive condensates alters the partitioning of a wider network of RBPs, which may affect, in multiple ways, protein translation. While under physiological conditions FMRP is in an equilibrium between a diffuse and a condensed state, increased cytoplasmic levels of FUS due to ALS mutations promote the phase separation of both proteins (Fig. 5, top). We find that wild-type FUS has a critical role in FMRP association with the translational machinery, but because this effect is comparable in both mutant FUS expression and in loss of FUS, it cannot be accounted for the translation repression detected only in mutant FUS–expressing MNs. Instead, our data support a model where increased FMRP condensate formation, induced by mutant FUS, reduces the availability of FMRP-bound RNAs, ultimately altering their translation (Fig. 5). It was recently shown that an altered cross-regulation between FUS, FMRP, and the RBP HuD results in an aberrant axonal phenotype in FUS-ALS models (*52*). It is therefore likely that FUS and FMRP and possibly other LCR-containing RBPs with a similar biophysical behavior form a wider network and alterations in their cytoplasmic localization can influence their LLPS behavior. This would result in the generation of heterogeneous condensates in which the RNAs that are bound by RBPs present in the condensates are sequestered and their translation inhibited, with a major impact on overall neuronal functionality. FUS mislocalization and the aberrant phase separation of the RBP network can therefore alter neuronal function via a misregulation of finely tuned translational requirements, possibly contributing to ALS pathogenesis and ultimately affecting MN survival.

## MATERIALS AND METHODS

### Animals

∆14 *Fus* mice (B6N;B6J-Fus^tm1Emcf/H^, MGI (Mouse Genome Informatics) MGI:6100933) were previously described (*13*). *Fus* knockout mice were obtained from the Mouse Knockout Project [Fustm1(KOMP)Vlcg]. HB9::GFP (green fluorescent protein) [B6.Cg-Tg(Hlxb9-GFP)1Tmj/J], choline acetyltransferase (ChAT)–internal ribosomal entry site (IRES)–Cre [B6;129S6-*Chattm2(cre)Lowl*/J], and RiboTag (B6N.129-*Rpl22tm1.1Psam*/J) mice were obtained from the Jackson laboratory. All mouse lines were backcrossed onto C57BL/6 J animals for more than five generations. Both ∆14 *Fus* knockin and *Fus* knockout animals were maintained in heterozygosity, because homozygous mice die perinatally. Both mouse lines were crossed with heterozygous HB9::GFP mice when required.

For RiboTag experiments, both RiboTag and ChAT-Cre homozygous mice were crossed with either *Fus*^∆*14/+*^ or *Fus^+/−^* animals. Double transgenic mice were subsequently crossed to obtain experimental progeny.

All experiments were carried out following the guidelines of the UCL (University College London) Queen Square Institute of Neurology Genetic Manipulation and Ethics Committees and in accordance with the European Community Council Directive of 24 November 1986 (86/609/EEC). All procedures for the care and treatment of animals were carried out under license from the U.K. Home Office in accordance with the Animals (Scientific Procedures) Act 1986 Amendment Regulations 2012 and were approved by the UCL Queen Square Institute of Neurology Ethical Review Committee.

### Primary MN preparation

E12.5-14.5 embryos for ventral horn cultures were obtained from heterozygous *Fus*^∆*14/+*^ or *Fus^−/+^* mice (with or without the HB9::GFP transgene). Briefly, embryos were euthanized, a sample of the tail was used for genotyping, and the body was maintained in ice-cold Hibernate-E media supplemented with B27. Spinal cords of the correct genotype were then dissected, meninges were removed, and dorsal horns were resected. Spinal cord ventral horns were incubated in 0.025% trypsin for 10 min at 37°C. Trypsin was then removed, and the tissue was triturated in L15 media containing 0.4% bovine serum albumin (BSA) and deoxyribonuclease (DNase, 0.1 mg/ml). Neurons were pelleted through a 4% BSA cushion; resuspended in Neurobasal media (Thermo Fisher Scientific) containing 2% heat-inactivated horse serum, 1× B27, 1× GlutaMAX, 1× penicillin/streptomycin, 24.8 μM β-mercaptoethanol, brain-derived neurotrophic factor (BDNF, 1 ng/ml), glial cell line–derived neurotrophic factor (GDNF, 0.1 ng/ml), and ciliary neurotrophic factor (CNTF, 10 ng/ml); and immediately plated onto 13-mm coverslips, microfluidic chambers (MFCs), or 3-cm dishes that had been precoated first with poly-ornithine (10 μg/ml) and laminin (3 μg/ml). Neurons were maintained in culture in a humidified incubator at 37°C with 5% CO_2_ for 5 to 7 DIV. MNs were transfected at DIV 2 by magnetofection as previously described (*57*).

### iPSC maintenance and differentiation

Human iPSCs used in this study are the isogenic FUS^WT/WT^ and FUS^P525L/P525L^ lines that were derived and maintained as described (*58*) and differentiated into spinal MNs as described (*35*, *59*). Briefly, iPSCs stably transduced with a piggyBac vector carrying inducible Ngn2, Isl1, and Lhx3 (NIL) transgenes dissociated to single cells with Accutase (Thermo Fisher Scientific) and plated in Nutristem XF/FF medium (Biological Industries) supplemented with 10 μM ROCK inhibitor (Enzo Life Sciences) on Matrigel (BD Biosciences) at a density of 100,000 cells/cm^2^. The day after NIL expression was induced by adding doxycycline (dox) (1 μg/ml; Thermo Fisher Scientific) in Dulbecco’s modified Eagle’s medium (DMEM)/F12 medium [DMEM/F12 (Sigma-Aldrich), supplemented with 1× GlutaMAX (Thermo Fisher Scientific), 1× nonesssential amino acid (NEAA) (Thermo Fisher Scientific), and 0.5× penicillin/streptomycin (Sigma-Aldrich)]. The medium was changed every day. After 48 hours of dox induction, the medium was changed to Neurobasal/B27 medium [Neurobasal Medium (Thermo Fisher Scientific), supplemented with 1× B27 (Thermo Fisher Scientific), 1× GlutaMAX (Thermo Fisher Scientific), 1× NEAA (Thermo Fisher Scientific), and 0.5× penicillin/streptomycin (Sigma-Aldrich)], containing 5 μM DAPT and 4 μM SU5402 (both from Sigma-Aldrich). At day 5, cells were dissociated with Accutase (Thermo Fisher Scientific) and plated on Matrigel (BD Biosciences)–coated dishes or coverslips at the density of 100,000 cells/cm^2^. ROCK inhibitor (10 μM) was added for the first 24 hours after dissociation. Neuronal cultures were maintained in neuronal medium [Neurobasal/B27 medium supplemented with BDNF (20 ng/ml) and GDNF (10 ng/ml) (both from PreproTech) and l-ascorbic acid (20 ng/ml; Sigma-Aldrich)].

### MEFs and cell lines

MEFs were isolated from the embryonic tissue discarded from the primary MN preparation. Viscera were removed, and the remaining tissue was triturated with a blade and incubated in 0.25% trypsin for 20 min at 37°C. Trypsin was quenched, and cells were plated in DMEM (Thermo Fisher Scientific) containing 10% fetal bovine serum (FBS) and 1× penicillin/streptomycin. After 3 days in culture, cells were immortalized by transfection with Lipofectamine 3000 (Invitrogen) with the simian virus 40 (SV40) T antigen. After 5 to 7 passages at low density, the cultures presented a homogeneous cell population and started to grow steadily.

HeLa and MEF cells were maintained in DMEM containing 10% FBS and 1× penicillin/streptomycin and were maintained in a humidified incubator at 37°C with 5% CO_2_. Transfection was performed with Lipofectamine 3000 (Invitrogen) according to the manufacturer’s instructions, 0.3 to 0.6 μg of DNA per coverslip was used, and cells were analyzed 18 to 48 hours after transfection.

### Microfluidic chambers

MFCs were made with a Sylgard 184 silicone elastomer kit (Dow Corning) using epoxy resin molds previously designed in the laboratory (*60*). Once the MFCs were baked, reservoirs were cut, and the MFCs were mounted onto glass-bottom dishes (HBST-5040, WillCo Wells), precoated with poly-d-lysine (20 μg/ml). MFCs were then blocked with 0.8% BSA (BioXtra, Sigma-Aldrich) overnight, poly-ornithine (>3 hours), and last, laminin (overnight), before plating MNs. MFCs have 500-μm-long grooves that separate the somatic from the axonal compartment.

### Constructs

mCherry-FUS^513x^ (∆NLS) was gifted by D. Dormann, and pcDNA6-Flag-FUS was gifted by M.-D. Ruepp. pcDNA6-Flag-FUS^P525L^ was generated in the laboratory by PCR mutagenesis. pBABE-puro SV40 Large T antigen was a gift from T. Roberts (Addgene plasmid no. 13970; http://n2t.net/addgene:13970; RRID:Addgene_13970).

### Antibodies

The following antibodies were used: anti-FUS N-terminal [western blot (WB) 1:5000; catalog no. NB100-565, Novus Biologicals], anti-FUS C-terminal [immunofluorescence (IF) 1:300, WB 1:5000; catalog no. NB100-562, Novus Biologicals], anti-FUS (IF 1:400; catalog no. sc-47711, Santa Cruz Biotechnology), anti-∆14 [IF 1:300 (*13*)], β3-tubulin [1:1000; catalog no. 801202 (BioLegend); 1:500; catalog no. 302 306 (SySy); and 1:2000; catalog no. 119-154886 (Raybiotech)] anti-GFP (IF 1:1000; catalog no. GFP1011, Aves Labs), anti-FMRP (IF 1:300 and WB 1:1000; catalog no. ab17722, Abcam), anti-SMN1 (IF 1:300 and WB 1:1000; catalog no. 610646, BD Biosciences), anti-Flag M1 (IF 1:500; catalog no. F3040, Sigma), anti-G3BP1 (1:200; catalog no. 611126, BD Biosciences), anti-RPL26 (IF 1:800 and WB 1:2000; catalog no. ab59567, Abcam), anti-RPS6 (WB 1:1000; Cell Signaling Technology), anti-LAMP1 (IF 1:300; catalog no. ab25245, Abcam), anti–glyceraldehyde-3-phosphate dehydrogenase (GAPDH) (WB 1:5000; mab374, Millipore), anti-HA [WB 1:3000 and immunohistochemistry (IHC) 1:100; catalog no. H6908, Sigma-Aldrich], and anti-ChAT (IHC 1:100; Chemicon). Alexa Fluor–conjugated secondary antibodies were from Invitrogen (1:1000) or Jackson ImmunoResearch (1:500).

### Immunofluorescence

Cells were fixed in paraformaldehyde (PFA) solution [4% PFA and 4% sucrose in phosphate-buffered saline (PBS)] for 15 min at room temperature (RT). Samples were then permeabilized and blocked in a solution containing 10% HRS (Horse serum), 0.5% BSA, and 0.2% Triton X-100 in PBS for 15 min. Primary antibodies were diluted in blocking solution (10% HRS and 0.5% BSA in PBS) and incubated for 1 hour at RT. Secondary antibodies were diluted in blocking solution (10% HRS and 0.5% BSA in PBS) and incubated for 1 hour at RT.

Coverslips were mounted using Mowiol or Fluoromount-G (Thermo Fisher Scientific); MFCs were mounted with Ibidi mounting media. Imaging was carried out using a Zeiss LSM 780 inverted confocal microscope with a 40× oil immersion lens with 1.3 numerical aperture or with a Zeiss LSM 710 inverted confocal microscope with a 63× oil immersion lens with 1.4 numerical aperture. Images were digitally captured using ZEN 2010 software and analyzed using Fiji (ImageJ).

### Immunohistochemistry

For immunohistochemical analysis, 3-month-old mice were perfused with saline, followed by 4% PFA solution. Spinal cords were dissected, incubated in 20% (w/v) sucrose, embedded in Tissue-Tek optimum cutting temperature compound (OCT) (Sakura Finetek, 4583), and sectioned with an OTF cryostat (Bright Instruments). Slices were mounted on microscope slides, and sections were encircled with a hydrophobic barrier pen (Dako, S2002), permeabilized by three 10-min washes with 0.3% Triton X-100 in PBS, and blocked for 1 hour in 10% BSA and 0.3% Triton X-100 in PBS. Samples were then probed with primary antibodies overnight; the samples were then washed three times before incubation with secondary antibodies for 1 hour. Slides were then washed, mounting media were added, and samples were covered with 22 mm by 50 mm cover glass.

### Cellular translation assay

AHA labeling assays were carried out as previously described (*61*). Briefly, neurons were incubated in a neuronal methionine-free media consisting of methionine and cysteine-free DMEM supplemented with 0.26 mM l-cysteine, 0.23 mM sodium pyruvate, 10 mM HEPES (pH 7.4), 0.067 mM l-proline, 0.674 μM zinc sulfate, 5 nM B12, 1× GlutaMAX, 1× B27, 1× penicillin/streptomycin, BDNF (1 ng/ml), GDNF (0.1 ng/ml), and CNTF (10 ng/ml) for 30 min before the addition of 2 mM AHA or vehicle control for 30 min. Anisomycin (40 μM) was preincubated for 20 min and coincubated with AHA. Neurons were then fixed and permeabilized, and AHA was labeled by click chemistry using the Click-iT Cell Reaction Buffer Kit with an Alexa Fluor 555 alkyne (1 μM) following the manufacturer’s instructions.

### Proximity ligation assays

PLA was performed with Duolink In Situ Orange PLA reagents according to the manufacturer’s protocol (Sigma-Aldrich).

### Image analysis

Images were analyzed using Fiji (ImageJ). Digital deconvolution was performed using the following plug-ins: “Diffraction PSF 3D” was used to generate a theoretical point spread function for each wavelength, and “Parallel spectral deconvolution 2D” was used for the generation of the deconvolved image. Puncta number was quantified manually using the “Cell Counter” plug-in. SynPAnal (*62*) was used to quantify axonal puncta size. PLAs were analyzed by using Fiji particle analysis and PLA puncta with sizes comprising 0.05 to 3.00 μm were quantified. In translation assays, MNs were selected by HB9::GFP expression and imaged. The AHA–Alexa Fluor 555 signal within MN cell bodies was quantified as mean fluorescence intensity. All analyses were performed on blinded samples.

### Live imaging and analysis

Live axonal transport assays were performed in motor neuronal cultures grown in MFCs at DIV 7 to 8 as previously described (*63*). Briefly, for signaling endosomes and mitochondria transport assays, neurons were incubated with either 30 nM Alexa Fluor 555–HcT or 125 nM MitoTracker Deep Red (Molecular Probes, Invitrogen) for 30 min at 37°C. Cells were then washed, and new MN media containing 20 mM HEPES-NaOH (pH 7.4) were added. After 15 min, transport was assessed at 37°C using an inverted Zeiss LSM 780 microscope equipped with a Zeiss 40×, 1.3 numerical aperture differential interference contrast Plan-Apochromat oil immersion objective. Images were taken at 2 Hz for 2 to 4 min. Cargoes were tracked using the Fiji plug-in TrackMate (*64*), and data analysis was performed in R.

### Western blotting and coimmunoprecipitation

MN cultures were lysed in radioimmunoprecipitation assay buffer (RIPA) [50 mM tris-HCl (pH 7.5), 150 mM NaCl, 1% NP-40, 0.5% sodium deoxycholate, 0.1% SDS, 1 mM EDTA, 1 mM EGTA, Halt phosphatase, and protease inhibitor cocktail (Thermo Fisher Scientific)], incubated on a rotating wheel at 4°C for 1 hour, and then nuclei and cellular debris were spun down at 20,000*g* for 10 min. Supernatants were collected, Laemmli buffer was added, and samples were denatured at 98°C for 5 min.

For coimmunoprecipitation (co-IP) assays, E12.5-14.5 brains were used. Samples were homogenized in lysis buffer [20 mM HEPES (pH 7.4), 150 mM NaCl, 10% glycerol, Halt phosphatase, and protease inhibitor cocktail] and incubated on a rotating wheel at 4°C for 1 hour; nuclei and cellular debris were spun down at 20,000*g* for 20 min. Supernatants were collected, 1 mg of protein lysate was used per co-IP, and protein of interest was immunoprecipitated with 2 μg of antibody or appropriate immunoglobulin G control overnight. Protein A Sepharose beads (Sigma-Aldrich) were used to purify the antibody/protein complex; precipitates were washed three times before being eluted in Laemmli buffer.

Samples were separated on precast 4 to 15% Mini-PROTEAN TGX Stain-Free protein gels (Bio-Rad) and transferred onto a polyvinylidene difluoride membrane using a semidry Trans-Blot Turbo system (Bio-Rad), or NuPAGE 4 to 12% bis-tris protein gels were used, and proteins were blotted onto a nitrocellulose membrane using a Novex system (GE Healthcare). Western blots were developed with a Classico substrate (Millipore) and detected with a ChemiDoc imaging system (Bio-Rad). Densitometric quantification of bands was carried out using the software Image Lab (Bio-Rad).

### Polysome profiling

Cytoplasmic lysates from frozen E17.5 brains were prepared as described previously (*29*). Tissue was pulverized in a mortar under liquid nitrogen. The tissue powder was dissolved in 10 mM tris-HCl (pH 7.5), 10 mM NaCl, 10 mM MgCl_2_, 1% Triton X-100, 1% sodium deoxycholate, RiboLock RNase Inhibitor (0.4 U/ml; Thermo Fisher Scientific), 1 mM dithiothreitol, cycloheximide (0.2 mg/ml), and DNase I (5 U/ml; Thermo Fisher Scientific). Following a first centrifugation step for 1 min at 14,000*g* at 4°C to remove tissue debris, the supernatant was centrifuged for 5 min at 14,000*g* to pellet nuclei and mitochondria. Cleared supernatants were then loaded on a linear 15 to 50% sucrose gradient in 10 mM tris-HCl (pH 7.5), 100 mM NaCl, and 10 mM MgCl_2_ and ultracentrifuged in a SW41Ti rotor (Beckman) for 1 hour and 40 min at 40,000 rpm at 4°C in a Beckman Optima LE-80 K ultracentrifuge. After ultracentrifugation, gradients were fractionated in 1-ml volume fractions with continuous monitoring absorbance at 254 nm using an ISCO UA-6 ultraviolet detector. Proteins were extracted from each sucrose fraction of the profile using the methanol/chloroform protocol and solubilized in a sample buffer.

### Cloning and purification of MBP-FUS and mutants

MBP-FUS was a gift from N. Fawzi (Addgene, plasmid no. 98651; http://n2t.net/addgene:98651; RRID:Addgene_98651). The P525L FUS point mutation and the Δ14 mutant version of FUS were generated via site-directed mutagenesis using MBP-FUS. The amino acid sequence at the C terminus of the Δ14 mutant is “KAPKPDGPGGGPGGSHMGVSTDRIAGRGRIN*”.

MBP-FUS and mutants were expressed in *Escherichia coli* BL21 DE3 cells with rare codons for R, I, P, and L using chloramphenicol and kanamycin for selection. Following cell lysis by sonication, the protein was purified by nickel-affinity chromatography. The lysis buffer used contained 20 mM sodium phosphate, 500 mM NaCl, 5 mM β-mercaptoethanol, and 20 mM imidazole (pH 7.4). One Complete Protease Inhibitor tablet (Sigma-Aldrich) was added to the lysate from 2 liters of growth. The column was washed with the same buffer supplemented with 40 mM imidazole. Protein was eluted in lysis buffer with 400 mM imidazole. The protein was then further purified using gel filtration chromatography with a buffer containing 50 mM tris-HCl (pH 7.6), 150 mM NaCl, 5 mM β-mercaptoethanol, and 1 mM EDTA.

### Protein expression and purification of FMRP–C-terminal

The low-complexity disordered region of human FMRP_445–632_ (referred to as FMRP_LCR_) was expressed and purified as previously described (*23*). Briefly, His-Small Ubiquitin-like Modifier (SUMO)-FMRP was transformed into *E. coli* BL21-CodonPlus(DE3) RIL cells and grown at 37°C in LB. Protein expression was induced with 0.5 mM isopropyl-β-d-thiogalactopyranoside (IPTG) at an OD_600_ (optical density at 600 nm) ~ 0.6 and grown overnight at 25°C. Cells were harvested and lysed in lysis buffer containing 6 M guanidinium chloride (GdnHCl), 50 mM tris-HCl (pH 8.0), 500 mM NaCl, 20 mM imidazole, and 2 mM β-mercaptoethanol. Harvested cells were sonicated for 4.5 min (2 s on, 1 s off) and centrifuged. The supernatant of the lysate was then purified by nickel-affinity chromatography equilibrated with the lysis buffer. The column was washed in lysis buffer without 6 M GdnHCl and then eluted in buffer containing 50 mM tris-HCl (pH 8.0), 500 mM NaCl, 300 mM imidazole, and 2 mM β-mercaptoethanol. The His-SUMO tag was cleaved with ubiquitin-like protease, while dialyzed against cleavage buffer (50 mM tris-HCl, 150 mM NaCl, 20 mM imidazole, and 2 mM β-mercaptoethanol at pH 7.4) overnight at 4°C. FMRP was separated from the His-SUMO tag by nickel-affinity chromatography following the same steps described above. The fractions containing FMRP were collected, and successful separation of FMRP from the His-SUMO tag was verified with SDS–polyacrylamide gel electrophoresis (SDS-PAGE) gel. FMRP was concentrated and further purified using gel filtration chromatography with a buffer containing 4 M GdnHCl, 50 mM tris-HCl (pH 8.0), 500 mM NaCl, and 2 mM β-mercaptoethanol.

### Fluorescence protein labeling

An Alexa Fluor 647 fluorescent dye was added to the only cysteine (C584) in FMRP_445–632_ via a maleimide linkage following the manufacturer’s instruction with slight modifications. First, FMRP was dialyzed into a buffer containing 50 mM tris-HCl (pH 7.5), 100 mM NaCl, and 4 M GdnHCl. To ensure that any residual reducing agents were removed, the protein was desalted using a Hi-Trap desalting column (GE Healthcare). After desalting, the protein sample was immediately reacted with 5× Alexa Fluor 647 (Thermo Fisher Scientific) maleimide dye. The reaction was incubated overnight at 4°C and quenched with an excess of reducing agent [dithiothreitol (DTT)] the following day. To remove any unreacted dye, the protein was passed through a Hi-Trap desalting column (GE Healthcare) and an S75 gel filtration column equilibrated in buffer containing 50 mM tris-HCl (pH 7.5), 100 mM NaCl, 4 M GdnHCl, and 2 mM DTT. Successful dye separation was confirmed by running the protein sample on an SDS-PAGE gel and then visualizing any remaining free dye with a fluorescence reader, ChemiDoc MP System (Bio-Rad). Labeled proteins were either frozen or dialyzed into specific assay buffers.

### RNA preparation

Sc1 RNA (GCUGCGGUGUGGAAGGAGUGGUCGGGUUGCGCAGCG) was purchased from Sigma-Aldrich as lyophilized samples. Stocks (100 μM) were reconstituted in water and stored at −20°C. Working stocks were diluted into specific assay buffers.

### Turbidity measurements

For turbidity measurements, OD_600_ of MBP-FUS was obtained using a SpectraMax i3× multimode plate reader (Molecular Devices) at 25°C. The samples were prepared by mixing varying concentrations of MBP-FUS with 0.5 μM TEV protease in a buffer containing 25 mM tris-HCl (pH 7.4), 150 mM KCl, and 2 mM DTT. Samples were equilibrated for 5 min before reading the turbidity. Turbidity was measured at intervals of 35 s for a total of 20 min. The change in turbidity was calculated from the slope (∆ absorbance/min) from 0 to 5 min. Apparent *C*_sats_ are calculated as previously described (*48*).

### In vitro co-LLPS assays

In vitro phase separation assays of LCRs of FUS and FMRP were performed using ^FITC^FUS_LCR_ (50 μM) and ^Alexa647^FMRP_LCR_ (50 μM) in the presence of 1 μM sc1 RNA in a buffer containing 25 mM sodium phosphate (pH 7.4), 50 mM KCl, and 2 mM DTT. For partitioning assays using full-length FUS proteins (FUS^WT^, FUS^P525L^, and FUS^Δ14^), MBP-FUS (10 μM) phase separation was induced by TEV protease (0.5 μM) cleavage before the addition of ^Alexa647^FMRP_LCR_ (1 μM) and sc1 RNA (0.5 μM) in a buffer containing 25 mM sodium phosphate (pH 7.4), 150 mM KCl, and 2 mM DTT.

### Fluorescence microscopy of phase-separated samples

Fluorescence images of phase-separated droplets were imaged on a confocal Leica DMi8 microscope equipped with a Hamamatsu C9100-13 electron multiplying charge-coupled device (EM-CCD) camera with a 63× objective. Alexa Fluor 647 fluorescence was detected using a 637-nm laser, and FITC fluorescence was detected using a 491-nm laser. In experiments with MBP-FUS and MBP-FUS mutants, samples were incubated with TEV protease for 10 min before imaging. All phase-separated droplets were imaged on a 96-well glass plate (Eppendorf). Two- or threefold concentrated protein or RNA samples were prepared to account for the dilution in mixing with other components to achieve desired final concentrations. Note that no molecular crowding reagents were used. Images represent droplets settled to the bottom of the plate. The images were processed using Volocity (PerkinElmer) and ImageJ.

### In vitro partitioning assay

To determine the partitioning of FMRP, images of droplets with the addition of 5% ^Alexa647^FMRP_LCR_ were acquired as described above and analyzed with ImageJ. An image of the buffer in the absence of any protein was used to subtract any background artifacts. In ImageJ, masks were defined using the Otsu threshold method while applying several criteria to the particle picking algorithm: droplets are required to have a radius greater than 1 μm and with the circularity of 0.5 to 1.0. The intensity of the bulk background solution is defined as the mean intensity within a circular region of interest with a diameter of 5 μm that does not contain any phase-separated droplets. Fluorescence enrichment ratios were calculated from the ratio of the mean fluorescence intensity (inside droplet)/mean fluorescence intensity (background outside of droplet). Droplets were randomly imaged, and measurement represents three independent experiments.

### In vitro translation assay

In vitro translation rates represent the increase in luminescence as a function of time using a standard rabbit reticulocyte lysate system (Promega) with luciferase mRNA (Promega). Manufacturer’s instructions were followed with a few modifications. Briefly, each reaction (30 μl) contains 12.6 μl of rabbit reticulocyte lysate, 0.5 μl of luciferase mRNA (1 mg/ml), 0.3 μl of amino acid mixture minus leucine (1 mM), 0.3 μl of amino acid mixture minus methionine (1 mM), 2 μl of TEV protease (15 μM), and 14.3 μl of 5 μM protein (MBP-FUS/FMRP) or buffer [25 mM sodium phosphate (pH 7.4), 50 mM KCl, and 2 mM DTT). First, the reaction was incubated for 10 min, and then, end-point luminescence measurements were carried out in intervals of 10 min up to 50 min. Each end-point luminescence measurement contained 75 μl of luciferase substrate mixed with 2.5 μl of unpurified translation mixture measured in a white opaque 96-well plate (Corning 3990). A SpectraMax i3× multimode plate reader (Molecular Devices) at 25°C was used to detect the luminescence. The translation rates represent the line of best fit from the end-point luminescence readings as a function of time.

### RiboTag

The RiboTag method was performed as described previously (*65*) with modifications. Briefly, E17.5 spinal cords were homogenized using TissueRuptor (Qiagen) in a buffer [50 mM tris-HCl (pH 7.4), 100 mM KCl, 12 mM MgCl_2,_ and 1% NP-40] supplemented with cycloheximide (0.1 mg/ml), heparin (1 mg/ml), SuperaseIn RNase (Thermo Fisher Scientific). Lysates were cleared by centrifugation at 10,000*g* for 10 min, and 5% of the lysate was saved as input. To reduce nonspecific-binding protein G magnetic beads (Dynabeads, Thermo Fisher Scientific) were added to the lysate and incubated for 2 hours at 4°C. Next, 5 μl of anti-HA antibody (Sigma-Aldrich) was added to the precleared lysate and incubated for 2 hours at 4°C. Later, 100 μl of beads slurry with 2 μl of SuperaseIn were added to the lysate followed by 2-hour incubation in the cold room. After precipitation, beads were washed five times in the wash buffer [300 mM KCl, 1% NP-40, 50 mM tris-HCl (pH 7.4), 12 mM MgCl_2_, and cycloheximide (0.1 mg/ml)]. Beads were eluted in Qiazol (Qiagen), and RNAs were isolated using the RNeasy Micro Kit (Qiagen). Ten percent of the beads were used for WB and eluted in Laemmli sample buffer (Thermo Fisher Scientific) supplemented with 100 mM DTT. The quality control of RNA was performed using TapeStation (Agilent).

### qPCR analysis of RiboTag samples

SuperScript IV VILO (Thermo Fisher Scientific) was used to reverse-transcribe RNA from input and IP samples (concentrations adjusted). Complementary DNA was used for qPCR analysis; expression of *Gapdh* was used as housekeeping control. Primers used for qPCR analysis are as follows: *Chat*, GCGTAACAGCCCAGGAGAG (forward) and TTGTACAGGCATCTTTGGGG (reverse); *Gapdh*, CAAGCTCATTTCCTGGTATGA (forward) and CTCTTGCTCAGTGTCCTTGCT (reverse); *HA-Rpl22*, GTGCCTTTCTCCAAAAGGTATTT (forward) and GTCATATGGATAGGATCCTGCATA (reverse); and *Pmp22*, GCCGTCCAACACTGCTACTC (forward) and GAGCTGGCAGAAGAACAGGA (reverse).

### RNA sequencing and analysis of RiboTag samples

Libraries were prepared using NEBNext mRNA Ultra II in the UCL Genomics facility and sequenced (75 base pairs single end) to an average depth of 18 million reads. Each sample was aligned to the *Mus musculus* (house mouse) genome assembly GRCm38 (mm10) with STAR (v2.4.2a) (*66*). Reads were coordinate-sorted and marked for PCR duplicates using Novosort (1.03.09). Gene expression was quantified using HTSeq using the Ensembl mm10 (v82) mouse transcript reference (*67*). Differential gene expression was calculated using DESeq2 (*68*) comparing the IP samples between the *Fus^−/−^* (*n* = 4) and *Fus*^∆*14/*∆*14*^ (*n* = 5) with the same number of their respective littermate controls, in two separate analyses. The significance level was set at a false discovery rate–adjusted *P* value of 10%. To compare the two analyses, each nominal *P* value was converted into a *z* score and given the sign of the log_2_ fold change. We defined condition-specific genes as having an adjusted *P* value of <0.1 in one condition and >0.1 in the other. A list of FMRP target genes was obtained from a published HITS (high-throughput sequencing)-CLIP experiment (*30*). Entrez IDs were converted to Ensembl IDs using g:Convert from the g:Profiler suite of tools (*69*). A list of FUS target peaks was obtained from a published iCLIP experiment (*32*). Peaks were annotated using annotatr (*70*) using gene models included in the TxDb.Mmusculus.UCSC.mm9.knownGene and org.Mm.eg.db R packages (*71*, *72*). FUS targets were filtered to only use peaks overlapping coding and UTR regions. GO terms analysis was performed in R using the enrichGO function from the clusterProfiler package (*73*).

### Statistical analysis

Unless otherwise stated, data were obtained using cells from at least three independent preparations, which are visualized in different shades of gray in the graphs. The number of cells studied is given in the figure legends. GraphPad Prism or R was used for statistical analysis. Normality of data distribution was tested using D’Agostino and Pearson normality test. One-way analysis of variance (ANOVA), followed by Dunnet’s post hoc test, was used for normally distributed data and multiple comparisons, while Sidak’s post hoc test was used for pairwise comparisons. Kruskal-Wallis, followed by Dunn’s post hoc test, was used for not normally distributed data and multiple comparisons. Friedman’s test, followed by Dunn’s post hoc test, was used to compare normally distributed paired samples. Individual differences were assessed using individual Student’s *t* tests. Data are shown as means ± SEM. Kolmogorov-Smirnov test was used in cumulative frequency analysis to test differences between targets and nontargets of FUS and FMRP.

## SUPPLEMENTARY MATERIALS

Supplementary material for this article is available at http://advances.sciencemag.org/cgi/content/full/7/30/eabf8660/DC1

## Appendix

View/request a protocol for this paper from *Bio-protocol*.
